# Relatives experience in moroccan psychiatric emergency

**DOI:** 10.1192/j.eurpsy.2023.1827

**Published:** 2023-07-19

**Authors:** A. Tounsi, B. Zineb, H. Chebli, F. Laboudi, A. Ouanass

**Affiliations:** Arrazi university psychiatric hospital, Sale, Morocco

## Abstract

**Introduction:**

In terms of care, family has a major role to play in the evolution of psychiatric illness.

Our aim in this work is to determine the family experience during the hospitalization of their sibling in the emergency room of the psychiatric university hospital Ar-Razi in Salé.

**Objectives:**

Our aim in this work is to determine the family experience during the hospitalization of their sibling in the emergency room of the psychiatric university hospital Ar-Razi in Salé.

**Methods:**

Our work was carried out with families of patients admitted to the emergency department of the psychiatric university hospital Ar-Razi in Salé.

The data collection was carried out with the help of a questionnaire including several items.

**Results:**

Sixty-five family members were included in this study. Their relatives hospitalized in the psychiatric emergency department were male in 70% of cases with an average age of 32.4 years.

- In 76% of the cases, the family member interviewed was the one who requested hospitalization;
- 55% of those interviewed were parents.

The analysis of relatives’ feelings during the hospitalization showed:

- Feelings of guilt were related to feelings of fear of exclusion and worries.
- Feelings of fear and exclusion were mostly expressed by mothers of patients hospitalized for the first time

At the end of the hospitalisation, 90% expressed relief, and 85% of family members were satisfied with their relative’s stay in the psychiatric emergency department.

**Conclusions:**

Recognizing families as units of care and understanding their situation and experiences facilitates the post-hospitalization care process. A well-informed family about mental illness and the types of therapeutic treatments available helps optimize the treatment.

**Disclosure of Interest:**

None Declared

